# Influenza A virus nucleoprotein is acetylated by histone acetyltransferases PCAF and GCN5

**DOI:** 10.1074/jbc.RA117.001683

**Published:** 2018-03-19

**Authors:** Dai Hatakeyama, Masaki Shoji, Seiya Yamayoshi, Rina Yoh, Naho Ohmi, Shiori Takenaka, Ayaka Saitoh, Yumie Arakaki, Aki Masuda, Tsugunori Komatsu, Rina Nagano, Masahiro Nakano, Takeshi Noda, Yoshihiro Kawaoka, Takashi Kuzuhara

**Affiliations:** From the ‡ Laboratory of Biochemistry, Faculty of Pharmaceutical Sciences, Tokushima Bunri University, Tokushima 770-8514, Japan,; § Division of Virology, Department of Microbiology and Immunology, Institute of Medical Science, University of Tokyo, Tokyo 108-8639, Japan,; ¶ Institute for Virus Research, Kyoto University, Kyoto, 606-8507, Japan,; ‖ PRESTO, Japan Science and Technology Agency, Saitama 332-0012, Japan,; ** Department of Special Pathogens, International Research Center for Infectious Diseases, Institute of Medical Science, University of Tokyo, Minato-ku, Tokyo 108-8639, Japan, and; ‡‡ Department of Pathobiological Sciences, School of Veterinary Medicine, University of Wisconsin-Madison, Madison, Wisconsin 53711

**Keywords:** acetylation, acetyltransferase, histone acetylase, influenza virus, influenza virus A, viral protein, GCN5, HAT, NP, nucleoprotein, PCAF

## Abstract

Histone acetylation plays crucial roles in transcriptional regulation and chromatin organization. Viral RNA of the influenza virus interacts with its nucleoprotein (NP), whose function corresponds to that of eukaryotic histones. NP regulates viral replication and has been shown to undergo acetylation by the cAMP-response element (CRE)–binding protein (CBP) from the host. However, whether NP is the target of other host acetyltransferases is unknown. Here, we show that influenza virus NP undergoes acetylation by the two host acetyltransferases GCN5 and P300/CBP-associated factor (PCAF) and that this modification affects viral polymerase activities. Western blot analysis with anti–acetyl-lysine antibody on cultured A549 human lung adenocarcinoma epithelial cells infected with different influenza virus strains indicated acetylation of the viral NP. A series of biochemical analyses disclosed that the host lysine acetyltransferases GCN5 and PCAF acetylate NP *in vitro*. MS experiments identified three lysine residues as acetylation targets in the host cells and suggested that Lys-31 and Lys-90 are acetylated by PCAF and GCN5, respectively. RNAi-mediated silencing of *GCN5* and *PCAF* did not change acetylation levels of NP. However, interestingly, viral polymerase activities were increased by the *PCAF* silencing and were decreased by the *GCN5* silencing, suggesting that acetylation of the Lys-31 and Lys-90 residues has opposing effects on viral replication. Our findings suggest that epigenetic control of NP via acetylation by host acetyltransferases contributes to regulation of polymerase activity in the influenza A virus.

## Introduction

In eukaryotic cells, gene expression is epigenetically regulated by several kinds of histone modifications (1, 2). Acetylation is one of the most important modifications for the epigenetic control of heterochromatin assembly and transcriptional activity (3). Acetylation levels in host histones were manipulated by infection with several viral species (4–7). Previous studies showed that several kinds of viral proteins were also reported to be targets of acetylation such as the Tat protein of HIV (8), the latency-associated nuclear antigen of Kaposi's sarcoma-associated *herpesvirus* (9) and the E2 protein of human papillomavirus (10). These proteins function as nonstructural proteins and their acetylation was important for their transcriptional activation. Therefore, they function as transactivators of transcription, not as structural proteins like nucleoprotein (NP)^3^ in the influenza A virus.

NP of the influenza A virus interacts with the viral RNA genome (vRNA), whose function corresponds to that of eukaryotic histones that interact with genomic DNA. NP receives multiple posttranslational modifications, which play crucial roles in regulating NP functions. Phosphorylation of NP inhibits its oligomerization and, consequently, ribonucleoprotein (RNP) activity and viral growth (11, 12). NP participates in modulating intracellular localization of RNP and itself by interacting with importin-α (13, 14), and SUMOylation and phosphorylation of NP control its trafficking between the nucleus and cytoplasm (15, 16). Furthermore, ubiquitination and deubiquitination of NP probably regulate the viral genome replication (17, 18). In addition to modifications on NP of influenza A virus mentioned above, acetylation on this viral protein was recently reported (19). They showed that eight lysine residues of NP were acetylated in HEK293 T cells whose acetyltransferase cAMP-response element (CRE)–binding protein (CBP) was co-expressed, and suggested that NP acetylation on three lysine residues effected the viral replication.

In this study, we succeeded in finding two acetyltransferases in host cells, GCN5 and PCAF, other than CBP, which acetylated NP at target lysine residues and consequently affected viral transcriptional activities. Mass spectrometry identified different candidate lysine residues that may have undergone acetylation by the two enzymes. Interestingly, viral transcriptional activities were increased by the RNAi against PCAF but were decreased by that against GCN5, suggesting that the different lysine residues targeted for acetylation caused these opposing results. Our findings provide insights to understand the epigenetic molecular mechanisms that regulate viral growth through posttranslational modifications.

## Results

### Identification of acetylated proteins in cells infected with influenza virus

**Figure 1.**
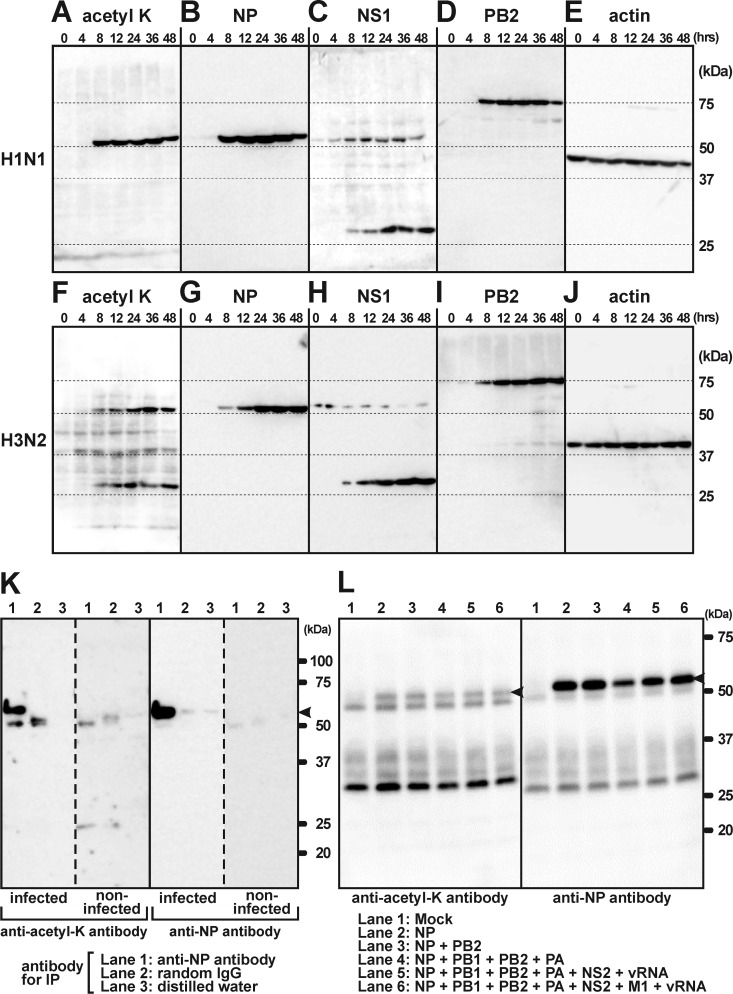
**Biochemical analyses using anti–acetyl-lysine antibody showed the acetylation of NP in the H1N1 (A/Puerto Rico/8/34) and H3N2 (A/Uruguay/716/2007) strains of influenza A virus.** *A* and *F*, a simple Western blot analysis of homogenized cultured cells using anti–acetyl-lysine antibody showed positive bands of around 50 kDa 8 h after infection with the H1N1 strain. The same bands were observed in cells infected by the H3N2 strain, together with bands at around 25 kDa. *B* and *G*, the positive bands at 50 kDa shown by anti–acetyl-lysine antibody in (*A*) and (*F*) were also detected by anti-NP antibody, suggesting that the acetylated protein was NP. *C* and *H*, Western blotting using anti-NS1 antibody suggested that the positive bands at 25 kDa shown by anti–acetyl-lysine antibody in (*F*) were NS1. This anti-NS1 antibody exhibited cross-reactions with NP. *D* and *I*, the molecular weight of PB2 did not correspond to the protein positively detected by anti–acetyl-lysine antibody. *E* and *J*, Western blotting using anti-actin antibody showed no differences in the loaded amount among samples. *K*, combined experiments of immunoprecipitation by anti-NP antibody and Western blotting by anti–acetyl-lysine antibody showed that the acetylated protein was NP (*arrowhead*). *L*, NP and other viral proteins were overexpressed in cultured cells. NP (*arrowheads*) individually overexpressed in cells (*lane 2*) was acetylated, suggesting that acetylation of NP occurred independently of other viral proteins.

To identify acetylated proteins during viral growth in host cells, we performed a Western blot analysis using anti–acetyl-lysine antibody. Cultured A549 human lung adenocarcinoma epithelial cells were infected with two different strains of influenza A virus (A/Puerto Rico/8/34 (H1N1) or A/Uruguay/716/2007 (H3N2)) and prepared for SDS-PAGE at 0, 4, 8, 12, 24, 36, and 48 h after infection. In cells infected by the H1N1 strain, a strong signal was detected as single bands of around 50 kDa in mass using the anti–acetyl-lysine antibody without any extra bands (Fig. 1*A*). By infecting cells with the H3N2 strain, two positive sets of bands of different sizes were strongly detected at around 50 kDa and 25 kDa, as well as proteins of host cells at around 37 kDa and 45 kDa (Fig. 1*F*). The viral titer of this strain was lower than that of the H1N1 strain. Therefore, it was suggested that the signal intensity of the acetylated NP of the H3N2 was not strong enough to eliminate the extra signals of host proteins. The two bands exhibiting strong signals at 50 kDa and 25 kDa were concluded to be viral proteins because they were both detected 8 h after infection (Fig. 1, *A* and *F*). To determine which viral proteins were acetylated, a Western blot analysis using antibodies for several viral proteins was performed. Strong signals were detected at around 50 kDa by anti-NP antibody in both strains of influenza virus (Fig. 1, *B* and *G*). In addition, bands were observed above the 25 kDa marker bands by anti-NS1 antibody (Fig. 1, *C* and *H*). Both expression and acetylation of NP and NS1 started 8 h after infection (Fig. 1, *A–C* and *F–H*). Acetylation of NS1 was limited to the H3N2 strain as reported previously (20), because positive signals on NS1 were observed only in the H3N2 strain, not the H1N1 strain (Fig. 1, *C* and *H*). We reported previously that PB2 interacted with acetyl-CoA (21), but acetylation of PB2 at around 75 kDa was not detected (Fig. 1, *A*, *D*, *F*, and *I*). Western blot analysis with anti-actin antibody showed that there was no difference in the amount of loaded samples among the lanes (Fig. 1, *E* and *J*). We used the other virus of the H3N2 strain (A/Hiroshima/52/2005) and obtained the same results of Western blot analysis with those of A/Uruguay/716/2007 (data not shown). These results suggested that NP of both strains was acetylated in cultured A549 cells.

### NP as an acetylated protein in host cells

Although Western blot analysis with anti–acetyl-lysine antibody suggested that there was acetylation of NP in host cells, this technique was not enough to specify which proteins were acetylated. To determine that this acetylated protein was indeed NP, a combination of immunoprecipitation using anti-NP antibody and Western blot analysis with anti–acetyl-lysine antibody was performed. Substitution of anti-NP antibody with random IgG or distilled water as negative controls failed to immunoprecipitate NP, showing that NP precipitation was specific (Fig. 1*K*). Western blot analysis with anti–acetyl-lysine antibody showed that NP was precisely acetylated in host cells (Fig. 1*K*). Overexpressed NP in cells was also acetylated (*lane 2* in Fig. 1*L*). Cotransfection of other viral proteins and vRNA with NP did not have any effect on its acetylation. These results showed that NP was acetylated in host cells without any interference from other viral proteins or from vRNA.

### Identification of acetyltransferases that acetylate NP

**Figure 2.**
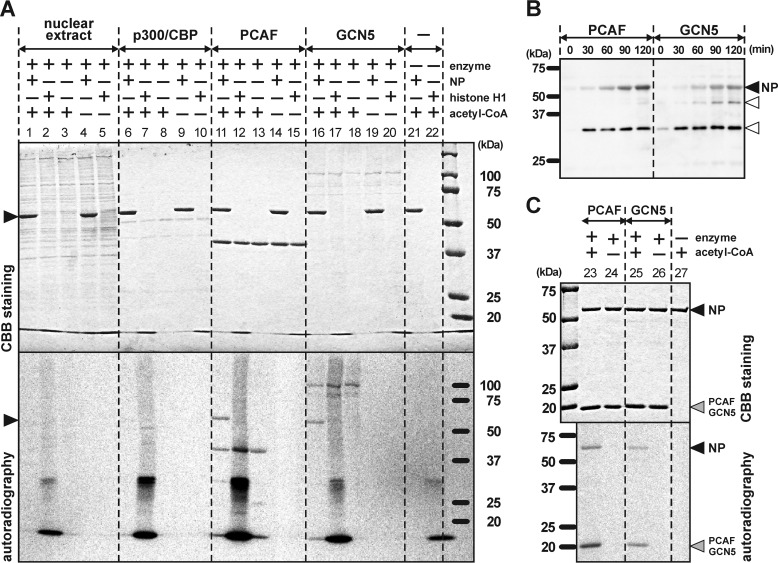
**Eukaryotic HATs acetylated NP *in vitro*.** *A*, the recombinant protein of NP derived from *E. coli* was acetylated by PCAF and GCN5, but not by the nuclear extract or by P300/CBP. Histone H1 was used as the positive control for acetylation. *B*, acetylation levels on the recombinant protein of the NP depended on incubation duration with the enzymes. The extra bands, from contamination of *E. coli* proteins, are indicated by *blank arrowheads. C*, RNP purified from virions of A/Puerto Rico/8/34 (PR8, H1N1) was incubated with PCAF and GCN5. NP constructing RNP was acetylated by PCAF and GCN5. Autoacetylation on the partial recombinant proteins of PCAF and GCN5 was also detected around the mass of 20 kDa. *Arrowheads* show the bands of NP. *Upper* and *lower* pictures of both panels show the results of Coomassie Brilliant Blue (*CBB*) staining and autoradiography, respectively.

To identify the enzymes in the host cells that acetylate NP, we performed an *in vitro* acetylation assay. The nuclear extract and recombinant proteins of three different acetyltransferases were incubated with the recombinant protein of NP (recombinant NP). The nuclear extract, which mainly contains P300/CBP with intrinsic histone acetyltransferase (HAT), and the recombinant protein of P300/CBP both did not acetylate NP (*lanes 1* and *6* in Fig. 2*A*). Histone H1 as a positive control was heavily acetylated, showing that the enzymatic activities of the nuclear extract and P300/CBP were conserved (*lanes 2* and *7* in Fig. 2*A*). On the other hand, the recombinant proteins of PCAF and GCN5 acetylated the NP (*lanes 11* and *16* in Fig. 2*A*). PCAF and GCN5 are grouped in the superfamily of GCN5-related *N*-acetyltransferases (GNAT) and their secondary and tertiary structures are highly similar to each other (22, 23). These results suggested that the NP was specifically acetylated by HATs belonging to the GNAT superfamily. Autoacetylation of PCAF (around 37 kDa) and GCN5 (around 100 kDa) was also detected. Acetylation on the NP by PCAF and GCN5 *in vitro* was also detected by Western blotting techniques using anti–acetyl-lysine antibody, and the intensity of the signal strengthened depending on the incubation time (Fig. 2*B*). Next, to investigate whether the NP that constructs part of the RNP was acetylated by PCAF and GCN5, these HATs were incubated with RNP purified from virions of the H1N1 strain (A/Puerto Rico/8/34). Acetylation of the NP within the RNP was clearly observed (*lanes 23* and *25* in Fig. 2*C*).

**Figure 3.**
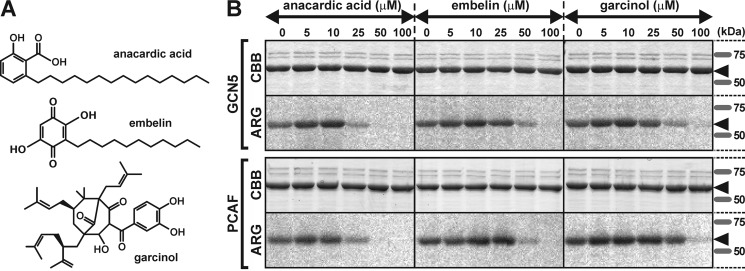
**Inhibition of acetylation of NP by HAT blockers *in vitro*.** *A*, chemical formulas of the HAT inhibitors anacardic acid, embelin, and garcinol. *B*, these chemicals blocked acetylation of NP in a concentration-dependent manner. Concentrations of anacardic acid greater than 25 μm inhibited acetylation. Embelin and garcinol showed acetylation inhibition at concentrations greater than 50 μm. *Arrowheads* show the bands of NP. *CBB* and *ARG* correspond to Coomassie Brilliant Blue staining and autoradiography, respectively.

PCAF and GCN5 contain Val-Lys-Gly (VKG) and Val-Arg-Gly (VRG) sites, respectively, which are required for acetyl-CoA interaction (21, 24). Results from a recent study on molecular docking simulations suggested that anacardic acid competes with acetyl-CoA for the VKG site in PCAF (25). Analogs of anacardic acid are also known to target the acetyl-CoA–binding site of other HATs, such as Tip60, and work as inhibitors of acetyltransferase activity (25). In addition to anacardic acid and its derivatives (26, 27), other types of natural chemicals have been reported to block HATs, such as embelin (28) and garcinol (Fig. 3*A*) (29). Therefore, we investigated whether these chemicals inhibit the acetylation on the NP by PCAF and GCN5. Anacardic acid exhibited the strongest effects among these three chemicals, with the acetyltransferase activities of PCAF and GCN5 being inhibited at concentrations of 25 μm (Fig. 3*B*). Embelin and garcinol were effective inhibitors at concentrations of 50 μm (Fig. 3*B*).

### Determination of acetylated lysine residues in NP

**Figure 4.**
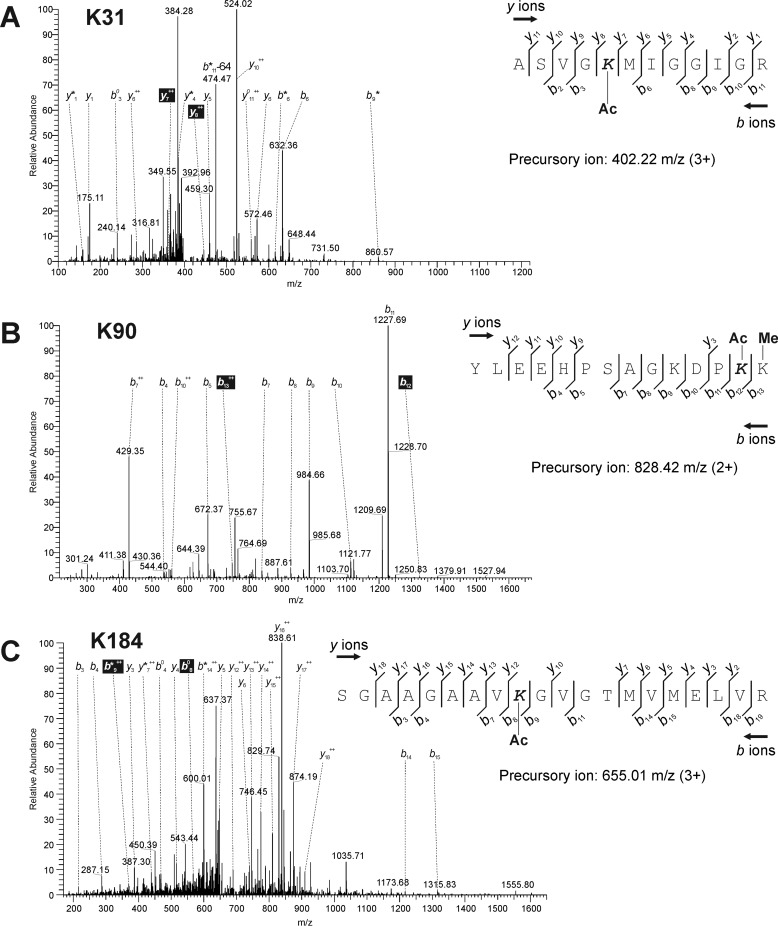
**LC-MS/MS analysis of influenza NP isolated from infected cells by immunoprecipitation.** *A–C*, base peak ion chromatogram derived from a 12-min separation of the digested NP peptides containing Lys-31 (*A*), Lys-90 (*B*), and Lys-184 (*C*). The elution time and representative *m*/*z* of the eluted peptides are indicated at the top of each peak. Formulas to calculate the molecular weights of acetylated lysine residues are shown in each panel. The observed *y* and *b* ions and fragment map are shown. *Ac* and *Me* mean an acetyl and a methyl group, respectively. *K31*, Lys-31; *K90*, Lys-90; *K184*, Lys-184.

To identify the acetylated lysine residues in host cells, NPs collected from infected cells by immunoprecipitation were analyzed by MS. The corresponding band was excised from SDS-PAGE gels and digested with trypsin. The digested peptide fragments were then analyzed using LC-MS/MS. All peptide sequence data matched the amino acid sequence of NP (A/Puerto Rico/8HY/1934(H1N1); GenBank^TM^ accession no. AKU37741). This technique identified three lysine residues that were candidates of acetylation targets within NP in infected cells (Table 1): Lys-31, Lys-90, and Lys-184. The peptide ASVGKMIGGIGR with the precursor *m*/*z* of 402.22 (*z* = 3) that matched NP residues 27–38 contained acetylated Lys-31 (Fig. 4*A*). However, this peptide was detected as an isotopic peak, suggesting that another modification might be present on this lysine residue (Table 1). The next peptide YLEEHPSAGKDPKK with the precursor *m*/*z* of 828.42 (*z* = 2), matching NP residues 78–91, harbored acetylated Lys-90 (Fig. 4*B*). Finally, in SGAAGAAVKGVGTMVMELVR with the precursor *m*/*z* of 655.01 (*z* = 3), matching NP residues 176–195, Lys-184 was acetylated (Fig. 4*C*). Ambiguous acetylation because of low scores by MASCOT search of MS was observed at Lys-103 (Table 1).

**Table 1:** **A summary of the detected acetyl-lysine residues in infected cells and the recombinant protein of NP incubated with PCAF and GCN5 *in vitro* and analyzed by LC-MS/MS** The peptide containing acetylated Lys-31 was detected as an isotopic peak, and another modification might occur on this lysine residue. Ambiguous acetylation because of low scores by MASCOT search of mass spectrometry was indicated by *asterisks*, such as Lys-77 and Lys-103. Among the three lysine residues detected as candidates of acetylation targets in host cells, acetylation of Lys-90 by PCAF and that of Lys-31 by GCN5 were not detected.

Infected cells	Recombinant NP incubated with pCAF	Recombinant NP incubated with GCN5
Lys-31	Lys-31	
	Lys-77*	
	Lys-87	Lys-87
Lys-90		Lys-90
	Lys-91	Lys-91
Lys-103*	Lys-103*	Lys-103*
Lys-184	Lys-184	Lys-184
	Lys-236	Lys-236
	Lys-273	Lys-273

**Figure 5.**
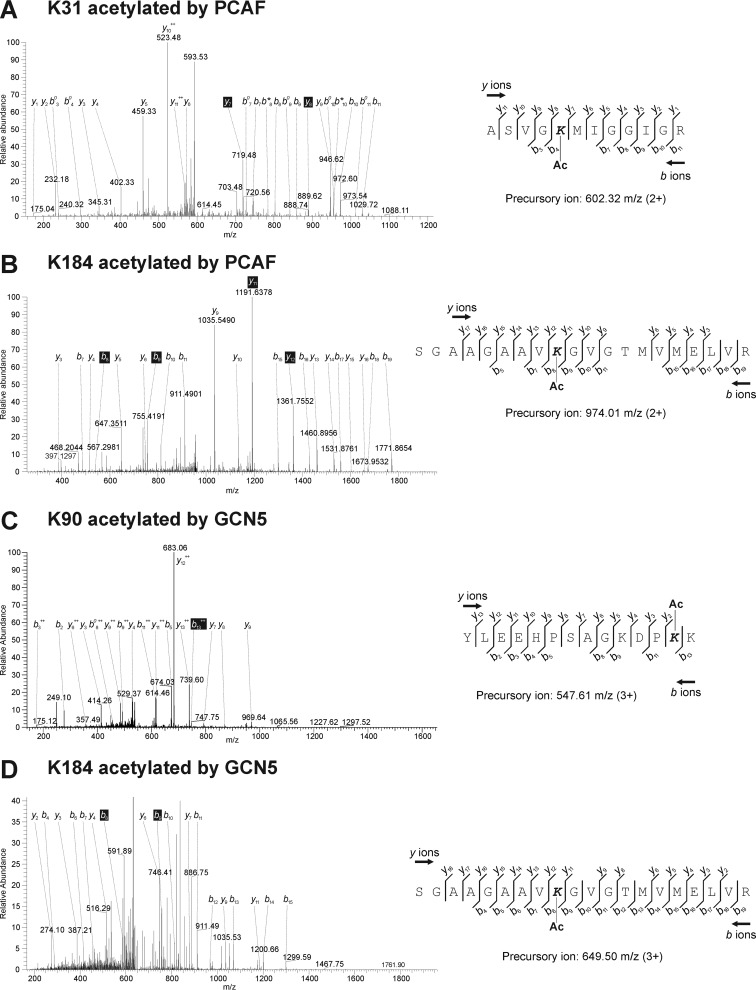
**LC-MS/MS analysis of recombinant protein of influenza NP incubated with PCAF and GCN5 *in vitro*.** *A–D*, base peak, ion chromatogram for a 12-min separation of the digested NP peptides containing Lys-31 (*A*) and Lys-184 (*B*) acetylated by PCAF and that contained Lys-90 (*C*) and Lys-184 (*D*) acetylated by GCN5. The elution time and representative *m*/*z* of the eluted peptides are indicated at the top of each peak. Formulas to calculate the molecular weights of acetylated lysine residues were shown in each panel. The observed *y* and *b* ions and fragment map are shown. *K31*, Lys-31; *K90*, Lys-90; *K184*, Lys-184.

To determine the target lysine residues that were acetylated by different acetyltransferases, we performed the same experiments using recombinant proteins of NP incubated with PCAF or GCN5 *in vitro*. Among the three lysine residues detected as candidates of acetylation targets in host cells (Fig. 4), acetylation of Lys-90 by PCAF and that of Lys-31 by GCN5 were not detected (Fig. 5, *A* and *C* and Table 1). The precursor *m*/*z* of each peptide and the corresponding amino acid sequences were 602.32 (ASVGKMIGGIGR with Lys-31 acetylated by PCAF) (Fig. 5*A*), 547.61 (YLEEHPSAGKDPKK containing Lys-90 acetylated by GCN5) (Fig. 5*C*), and 974.01 and 649.50 (SGAAGAAVKGVGTMVMELVR with Lys-184 acetylated by PCAF and GCN5, respectively) (Fig. 5, *B* and *D*). *In vitro* incubation of the recombinant proteins of NP and two kinds of HATs showed that other lysine residues, such as Lys-77, Lys-87, Lys-91, Lys-103, Lys-236, and Lys-273, together with three residues detected in the samples prepared from infected cells (Table 1). Acetylation on Lys-184, Lys-236, and Lys-273 was detected by incubation of both enzymes, suggesting that these residues were complementally acetylated.

These analyses showed that the precursor *m*/*z* of the peptide containing Lys-90 purified from infected cells (828.42 *m*/*z* in Fig. 4*B*) was different from that of the recombinant protein (547.61 *m*/*z* in Fig. 5*C*). In the peptides purified from the infected cells, the methylation on Lys-91 was detected together with acetylation on Lys-90 (Fig. 4*B*), and the molecular mass of this peptide was larger than that of the peptide from the recombinant protein in the mass of methyl group. Variable modifications, such as ubiquitination and methylation, on lysine residues in NP were also detected in infected cells (data not shown). Acetylation on Lys-77 and Lys-103 in the recombinant proteins was ambiguous because of low MASCOT scores (Table 1).

**Figure 6.**
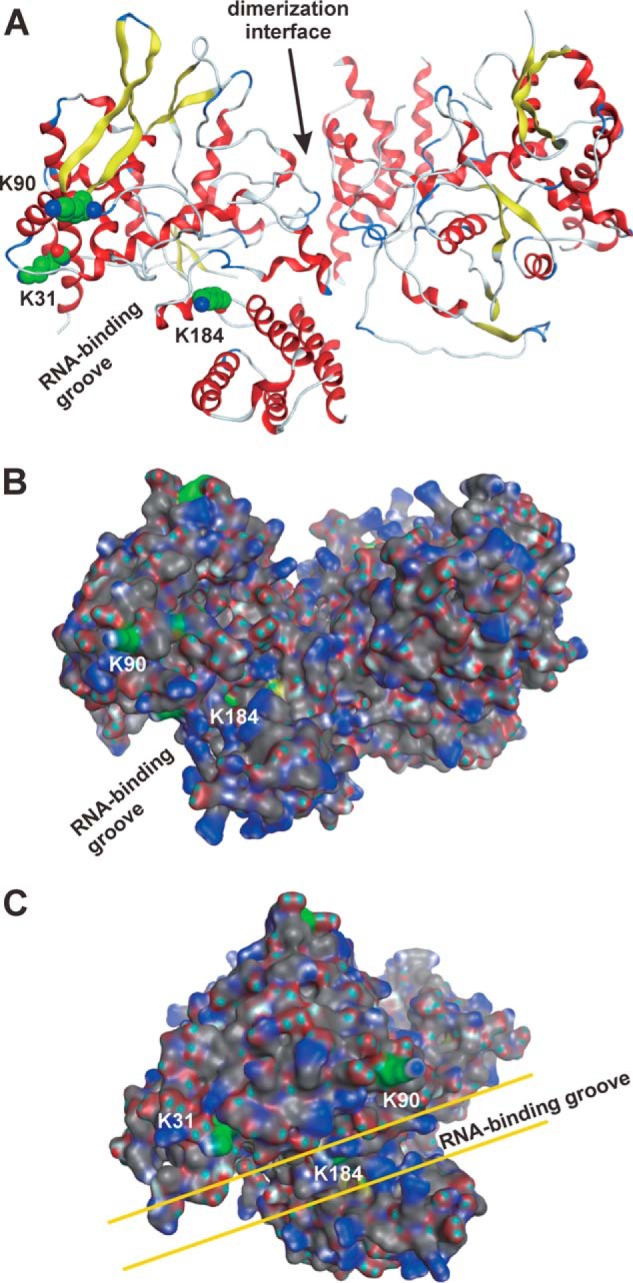
**Acetylated lysine residues in the tertiary structure of NP.** *A–C*, tertiary structure of influenza NP dimer (PDB ID: 4IRY) showed that candidate lysine residues for acetylation were concentrated on the surface of the RNA-binding groove. Ribbon structures (*A*) and surface structures (*B* and *C*) show the positions of target lysine residues using the MOE software. *A*, structures of the helix and strand are shown in *red* and *yellow*, respectively. The atoms of carbon and nitrogen in the side chains of the acetylated lysines are highlighted as *green* and *blue spheres*, respectively. *B* and *C*, the atoms of hydrogen, carbon, nitrogen, oxygen, and sulfur are shown in *cyan*, *gray*, *blue*, *red*, and *yellow*, respectively. The carbon atoms in the side chains of the acetylated lysines are shown in *green. Panel C* is the 90 degree–rotated structure of *panel B. K31*, Lys-31; *K90*, Lys-90; *K184*, Lys-184.

Especially focusing on the three lysine residues acetylated in cells, their positions in tertiary structure of NP were visualized by a molecular simulating system (PDB ID: 4IRY) (Fig. 6, *A–C*) (30). This result showed that all three residues were not localized in the interface for dimerization of the NP but exposed on the surface (Fig. 6*A*). All three lysine residues—Lys-31, Lys-90, and Lys-184—were localized in the RNA-binding groove (Fig. 6, *A–C*), suggesting that acetylation of these three lysine residues is involved in regulation of the interaction with vRNA, mRNA, and/or cRNA. Although Lys-184 was localized in an RNA-binding groove (Fig. 6*B*) (18), our molecular calculation using the Molecular Operating Environment (MOE) software showed that the side chain of Lys-184 (atoms indicated in *green* in Fig. 6, *B* and *C*) was exposed to the surface of the groove. This lysine residue was a target of ubiquitination, as reported previously (17, 18). Ubiquitination of Lys-184 in NP collected from infected cells was also notably detected in our experiments by MS (data not shown). Recently, the ubiquitination of Lys-184 was reported to be regulated by the E3 ubiquitin ligase, CNOT4 (18). CNOT4, with a molecular mass of 71 kDa, constructs huge Ccr4-Not complexes (31).

### Effects of NP acetylation by PCAF and GCN5 on viral transcriptional activity

**Figure 7.**
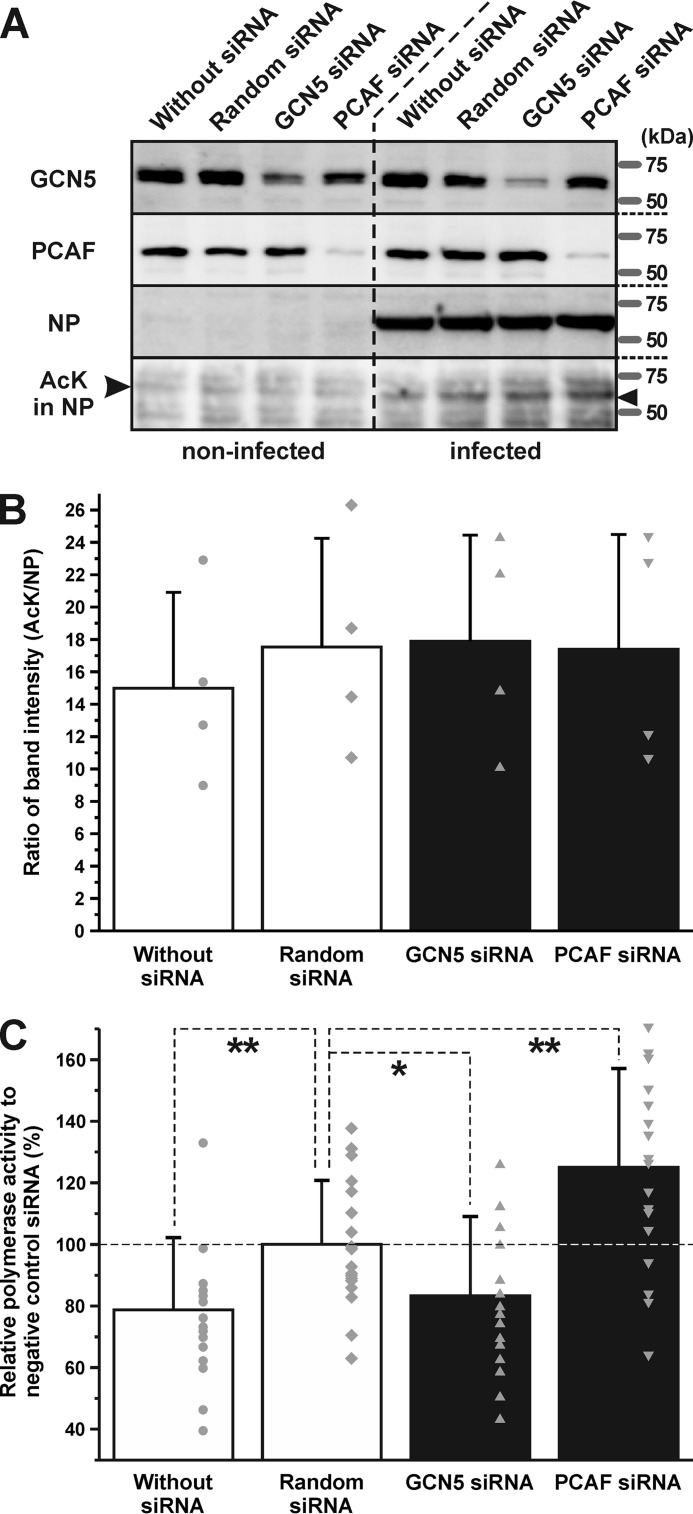
**Inhibition of expression of GCN5 and PCAF altered the polymerase activity of viral RdRp.** *A*, treatment of cultured A549 cells with the siRNAs of GCN5 and PCAF decreased the expression levels of these proteins. *B*, ratios of band intensities (AcK/NP) were calculated. There were no significant differences among the four groups. The data are represented as mean ± S.D. of all the individual data points and are indicated by *gray-colored circles*, *diamonds*, *triangles*, and *inverted triangles. C*, minigenome assays were performed using siRNA-treated cells. Transfection of random siRNA increased the relative polymerase activity significantly. Interestingly, compared with the cells injected with random siRNA, viral transcription increased with addition of PCAF siRNA, but decreased with GCN5 siRNA. The data are represented as mean ± S.D. of all the individual data points and are indicated by *gray-colored circles*, *squares*, and *triangles*. Relative polymerase activity was analyzed by one-way analysis of variance (ANOVA) followed by a Tukey's post hoc test (*, *p* < 0.05; **, *p* < 0.01).

In general, acetylation of histones causes alterations to the chromatin structure and stimulates transcriptional activity (2, 32). To investigate the biological significance of NP acetylation, transcriptional levels of viral RNAs were analyzed using cultured cells where GCN5 and PCAF expressions were inhibited by RNAi. Transfection of GCN5 and PCAF siRNAs were shown to reduce the expression levels of these enzymes (Fig. 7*A*). There were no changes in the amount and acetylation levels of NP (Fig. 7, *A* and *B*). We performed a minigenome assay to measure alterations to the polymerase activity after transfection of the siRNAs. Transfection of random siRNA increased the relative polymerase activity significantly (*p* < 0.01). However, interestingly, compared with the cells injected with random siRNA, viral transcription increased with the addition of PCAF-specific siRNA (*p* < 0.01), but decreased by that of GCN5 (*p* < 0.05) (Fig. 7*C*). These results suggested that GCN5 and PCAF regulated viral transcriptional activity in host cells.

## Discussion

In this work, a series of biochemical studies showed that the NP of the influenza A virus was acetylated in infected cells. *In vitro* experiments indicated that the host acetyltransferases, GCN5 and PCAF, were responsible for acetylating the NP of the influenza A virus. We succeeded in determining the candidate lysine residues for acetylation targets by MS. Treatment with siRNAs specifically targeted against PCAF and GCN5 did not alter the acetylation levels of NP in host cells. However, inhibition of PCAF and GCN5 expression by RNAi increased and decreased viral transcriptional activity, respectively, suggesting that the activities of these acetyltransferases are important for controlling viral growth. Generally, transcriptional activities in eukaryotic cells are regulated by acetylation of histone-constituting nucleosomes. In this study, we showed that the transcription of the influenza virus was regulated by acetylation of NPs, which interact with the vRNA of the influenza virus as well as with the histones that bind to genomic DNA. Here, we suggested that this epigenetic control also influences the transcriptional activity in the influenza virus.

Interestingly, the addition of siRNAs specific for PCAF and GCN5 had opposing effects on viral transcriptional activities (Fig. 7*C*). We suggested that this was because of different lysine residues being targeted for acetylation. Although mass spectrometric analyses using infected cells identified three lysine residues as candidates of acetylation targets (Fig. 4), the same experiment using incubated recombinant proteins of NP and eukaryotic acetyltransferases *in vitro* showed that Lys-31 and Lys-90 were candidates of acetylation by PCAF and GCN5, respectively (Fig. 5). Lys-31, Lys-90, and Lys-184 are localized on the surface of the RNA-binding groove. Acetylation of these lysine residues by GCN5 and PCAF is hypothesized to eliminate the positive charge and decrease the affinity of NP to vRNA and/or cRNA, thereby triggering transcriptional activity. However, Lys-31 and Lys-90 residues are localized in the opposite side of the RNA-binding groove (Fig. 6, *B* and *C*), suggesting that the acetylation of Lys-90 by GCN5 has a biological function different from that of Lys-31 by PCAF, which is involved in the defense responses of the host. Similar opposing effects of acetylation by PCAF and GCN5 were reported in tumor cells (33, 34). Acetylation on different lysine residues in the tumor repressor p53 caused a tumor-promoting role of GCN5 and a tumor-repressing role of PCAF (33). GCN5 is part of the coactivator complex SAGA (Spt-Ada-Gcn5-acetyltransferase) (35). GCN5 and TRRAP, other components of SAGA, interact with the MYC oncoprotein in mammalian cells (36, 37), which is the most important protein in cancers (38). In the case of influenza A virus infection, the expression level of MYC increased in primary human respiratory cells (39), and the transcription factor activity of the MYC-associated zinc finger (MAZ) protein was significantly higher (40). The same molecular cascade from GCN5 to MYC might be activated in tumor cells. On the other hand, in the process of tumor suppression, PCAF acetylated p53, increasing its DNA-binding ability (41). Previous reports showed that NP induced p53 signaling and apoptosis in infected cells (42). Moreover, p53 knockout mice were more susceptible to influenza A virus infection (43). Taken together, the molecular mechanisms of the opposite effects of GCN5 and PCAF in tumor and infected cells might be similar.

Similar to the influenza A virus, the hepatitis D virus harbors a negative-sense single-stranded RNA as its genetic material, which encodes for viral nucleocapsid proteins termed hepatitis D antigen (HDAg) (44), which have previously been reported to undergo acetylation (45). Similar to influenza NP, HDAg possesses histone-like functions and binds to nucleic acid. However, the function of this modification is still unclear. What is the biological significance of acetylation on influenza NP? Our data showing the localization of lysine residues of acetylation targets suggested that these lysine residues were involved in interaction with other factors except for influenza NP, such as bromodomain proteins which bind with acetyl-lysine residues (Fig. 6). Here, we suggest that the ATPase subunits of the two isoforms, SWI/SNF-related matrix-associated actin-dependent regulator of chromatin A2 (protein name SMARCA2, BRM, or SNF2L2) and A4 (protein name SMARCA4 or BRG1) are the key factors to answering this question. Previously, interactome screening was performed to identify targetable host factors, which were subsequently analyzed using the H1N1 strain (46), to discover that SMARCA2 and SMARCA4 interacted with NP. These proteins possess bromodomains for the interaction with acetylated lysine residues (47) and function as a core component of the mammalian SWI/SNF chromatin-remodeling complex (48). This complex has a multitude of subunits and performs fundamental roles in biological processes (49). The interaction between NP and SMARCA2/4 is suggested to be mediated by NP acetylation.

## Experimental procedures

### Cells

A549 cells were cultured in high-glucose Dulbecco's modified Eagle's medium (Wako) supplemented with 10% fetal bovine serum (Life Technologies), 50 units/ml penicillin and 50 μg/ml streptomycin (Life Technologies), and 4 mm l-glutamine, at 37 °C in the presence of 5% CO_2_.

### Western blot analysis to detect acetyl-lysine residues

A549 cells were seeded in a 24-well plate (2 × 10^5^ cells/well), and then infected with a multiplicity of infection (m.o.i.) of three influenza A virus strains (A/Puerto Rico/8/34 (H1N1), A/Uruguay/716/2007 (H3N2), and A/Hiroshima/52/2005 (H3N2)) at 37 °C under 5% CO_2_. After incubation for 0, 4, 8, 12, 24, 36, or 48 h, the cells were lysed in a buffer containing 125 mm Tris-HCl, pH 6.8, 5% SDS, 25% glycerol, 0.1% bromophenol blue, and 10% β-mercaptoethanol and boiled for 5 min. Samples of infected cells were separated by SDS-PAGE and transferred onto pieces of PVDF membranes (Immobilon-P; Merck Millipore Ltd.). To detect acetyl-lysine NP, NS1, PB2, or β-actin proteins, we used the mouse monoclonal anti–acetyl-lysine IgG (Clone 7F8, 1:200, Cayman Chemical), the mouse monoclonal anti-influenza A NP IgG (Clone FluA-NP 4F1, 1:500, SouthernBiotech), the rabbit anti-PB2 anti-serum (1:1000, originally produced in our lab), the goat polyclonal anti-influenza A NS1 IgG (vC-20, 1:200, Santa Cruz Biotechnology), and the rabbit monoclonal anti-β-actin IgG (Clone 13E5, 1:1000, Cell Signaling Technology) as the primary antibodies. The horseradish peroxidase–conjugated goat anti-mouse IgG (1:4000, SouthernBiotech), donkey anti-goat IgG (1:4000, sc-2020, Santa Cruz Biotechnology), and goat anti-rabbit IgG (1:5000, KPL) were used as the secondary antibodies. The luminescent signals were detected using Western Lightning ECL Pro (PerkinElmer Life Sciences).

### Immunoprecipitation and Western blotting

A549 cells seeded in a 6-well plate (1 × 10^6^ cells/well) were infected with m.o.i. of five A/Puerto Rico/8/34 at 37 °C under 5% CO_2_. After postincubation for 24 h, the cells were lysed in a buffer containing 50 mm Tris-HCl, pH 7.5, 150 mm NaCl, 0.5% Nonidet P-40, 1 mm EDTA, and the cOmplete ULTRA Tablets, Mini, EDTA-free, Protease Inhibitor Cocktail (Roche). The cell lysates were centrifuged at 15,000 rpm at 4 °C for 10 min and the supernatant was collected. Protein G magnetic beads (20-μl/sample, New England Biolabs) were incubated with monoclonal anti-NP antibody (1-μl/sample) at 4 °C for 30 min. The cell lysate was incubated with the antibody–protein G bead complex at 4 °C overnight. The beads were washed three times with lysis buffer, added to 30 μl SDS sample buffer, boiled at 95 °C for 5 min then separated by SDS-PAGE. Western blotting was performed using either the mouse monoclonal anti–acetyl-lysine IgG (Cayman Chemical) or the mouse monoclonal anti-influenza A NP IgG (SouthernBiotech), using the standard Western blotting procedure.

### Recombinant proteins of influenza NP, human PCAF and GCN5

The recombinant proteins of full-length influenza NP and HAT domains of human PCAF and GCN5 were synthesized in *Escherichia coli* and purified as previously published (21). The full-length cDNA for the NP of the influenza virus (A/Puerto Rico/8/34) was amplified by RT-PCR. The DNA sequences encoding the HAT domains of human PCAF (amino acid residues 493–653; GenBank accession no. NP_003875) (50) and GCN5 (amino acid residues 498–663; GenBank accession no. NP_066564) were partially amplified from cDNA of HaCaT cells. Nucleotide sequences of primers for cloning of NP were as follows: forward primer, 5′-GCT AGC ATG GCG TCC CAA GGC ACC AAA CGG-3′ (containing restriction site for NheI) and the reverse primer, 5′-CTC GAG TTA ATT GTC GTA CTC TGC ATT-3′ (containing restriction site for XhoI). Nucleotide sequences of primers for cloning of PCAF were as follows: the forward primer, 5′-GCT AGC GTA ATT GAA TTT CAC GTG GTT GGC-3′ (containing restriction site for NheI) and the reverse primer, 5′-GGA TCC TCA CCG TGG ATT TAG CTC ACA TCC-3′ (containing restriction site for BamHI). Nucleotide sequences of primers for cloning of GCN5 were as follows: forward primer, 5′-GCT AGC ATC GAG TTC CAT GTC ATC GGC-3′ (containing restriction site for NheI) and the reverse primer, 5′-GGA TCC TTA CTC CGT GTA GGG GAT GCG GGG-3′ (containing restriction site for BamHI). The underlined sequences are the restriction enzymes' recognition sites. Each PCR product was ligated into the pET28a(+) vector (Novagen). The recombinant proteins of full-length human GCN5 and the catalytic domain of human PCAF (Cayman Chemical) were also purchased.

### Acetylation assays using radioisotope-labeled acetyl-CoA

The procedures followed to perform this study were modified from a previous report (21). The recombinant proteins of NP (1 μg) were incubated with 1 μg of the recombinant protein of histone acetyltransferase (P300/CBP (Enzo Life Sciences), GCN5 or PCAF) and 7.4 kBq of [^14^C]acetyl-CoA (Perkin Elmer) at 30 °C for 2 h in buffer containing 50 mm Tris-HCl, pH 8.0, 10% glycerol, 1 mm DTT, and 10 mm sodium butyrate. To detect [^14^C] radioactivity, reactions were separated in 10% SDS-PAGE gels, after which an imaging plate was exposed to the gel for several days. Signals were detected using a fluoroimage analyzer (FLA-2000, Fujifilm). Anacardic acid (Sigma), garcinol (Enzo Life Sciences), and embelin (Sigma) prepared in DMSO were incubated with NP and a HAT for 30 min at 30 °C. The RNP was purified from virions of A/Puerto Rico/8/34 (PR8, H1N1) as described previously (51).

### Analysis of posttranslational modifications by LC-MS/MS

LC-MS/MS was performed using the NP in infected A549 cells as well as the recombinant NP. The NP in infected cells was collected by immunoprecipitation as mentioned above. Recombinant NP of the A/Puerto Rico/8/34/Mount Sinai strain (H1N1) (Novus Biologicals) was incubated with PCAF or GCN5. The amount of recombinant NP, the concentration of reaction solution, and the temperature and duration of incubation were the same as the acetylation assays, except for substitution of [^14^C]-marked acetyl-CoA with nonradioactive acetyl-CoA. Protein samples were separated in 10% SDS-PAGE gels. After staining with Coomassie Brilliant Blue, the NP bands were cut and extracted from the gels. Nano-LC tandem MS was performed as described previously (52). Next, in-gel digestion was performed with modified trypsin (sequencing grade, Promega) after the reduction and alkylation of each gel slice. The digested peptide mixtures were then fractionated by C18 reverse-phase chromatography (Advance UHPLC, AMR Inc.) and measured by a hybrid linear ion trap mass spectrometer (LTQ Orbitrap Velos Pro, Thermo Fisher Scientific) with Advance CaptiveSpray SOURCE (AMR). The molecular masses of the resulting peptides were searched against the nucleoprotein amino acid sequence using the MASCOT program. To identify nucleoprotein modifications, carbamidomethylation was set as the fixed modification and acetylation (+42.011), mono-methylation (+14.016), dimethylation (+28.031), and trimethylation (+42.047) were set as the variable modifications.

### Mapping of acetylated lysine residues in the tertiary structure of NP

Acetylated lysine residues were mapped in the tertiary structure of the dimer of the NP (PDB ID: 4IRY) (30) using the MOE software (Chemical Computing Group, Quebec, Canada). The X-ray crystallographic structure of the cap-binding domain of NP (PDB ID: 4IRY) was obtained from the Protein Data Bank.

### Western blotting and immunostaining using siRNA-treated cells

For Western blotting analyses (Fig. 6*A*), A549 cells (5 × 10^5^ per dish) seeded in a 6-well plate were transfected with 10 nm *Silencer*^TM^ Select Negative Control No. 2 siRNA (validated) (Ambion/Thermo Fisher Scientific) KAT2A: GCN5 (s5659, Ambion/Thermo Fisher Scientific), or KAT2B: PCAF (s16894, Ambion/Thermo Fisher Scientific) using Lipofectamine^TM^ RNAiMAX reagent (Thermo Fisher Scientific) and incubated for 48 h. The siRNA-treated cells were incubated at 37 °C with A/Puerto Rico/8/34 at m.o.i. 5 under 5% CO_2_ for 24 h. The cells were lysed in a buffer containing 50 mm Tris-HCl, pH 7.5, 150 mm NaCl, 0.5% Nonidet P-40, 1 mm EDTA, and a cOmplete ULTRA Tablet, Mini, EDTA-free Protease Inhibitor Cocktail (Roche). The cell lysates were centrifuged at 15,000 rpm at 4 °C for 10 min, and the supernatant was collected. Western blotting analysis was performed as described above. The intensity of the bands was measured using the software for image processing and Java and ImageJ.

### Minigenome assay using siRNA-treated cells

Procedures of culturing and siRNA treatment of cells were same as those of Western blotting analyses mentioned above. Minigenome assay based on the dual-luciferase system was performed as described previously (53–55). Briefly, the siRNA-treated A549 cells were transfected with pCA-NP, -PA, -PB1, and -PB2 or its mutants (0.2 μg each), pPolI/NP(0)Fluc(0) (0.2 μg), which express reporter vRNA encoding the firefly luciferase gene, and pRL-TK-*RLuc* (Promega, 0.2 μg), which express *Renilla* luciferase and are regulated by thymidine kinase (TK) promoter as an internal control. The NP ligated into the pCA plasmid was derived from the WSN strain (H1N1), and all lysine residues for acetylation targets were conserved. The luciferase activity was measured using the Dual-Glo Luciferase Assay System (Promega) at 24 h post transfection. Polymerase activity was calculated by normalizing the *Firefl*y luciferase activity to the *Renilla* luciferase activity. Polymerase activity of WT was set to 100%.

## Author contributions

D. H. resources; D. H., M. S., S. Y., R. Y., N. O., S. T., A. S., Y. A., A. M., T. Komatsu, R. N., M. N., T. N., and T. Kuzuhara data curation; D. H., M. S., S. Y., R. Y., N. O., S. T., A. S., Y. A., A. M., T. Komatsu, R. N., M. N., T. N., Y. K., and T. Kuzuhara formal analysis; D. H. funding acquisition; D. H., M. S., S. Y., R. Y., N. O., S. T., A. S., Y. A., A. M., T. Komatsu, R. N., M. N., and T. Kuzuhara investigation; D. H. writing-original draft; D. H., T. N., Y. K., and T. Kuzuhara writing-review and editing; S. Y. methodology; M. N., Y. K., and T. Kuzuhara supervision; M. N. validation; T. Kuzuhara conceptualization; T. Kuzuhara project administration.

## Footnotes

3 The abbreviations used are:

NP: nucleoprotein
vRNA: viral RNA
RNP: ribonucleoprotein
CBP: cAMP-response element (CRE)–binding protein
HAT: histone acetyltransferase
m.o.i.: multiplicity of infection
CBB: Coomassie Brilliant Blue.

## References

[1] Schiltz R. L., and Nakatani Y. (2000) The PCAF acetylase complex as a potential tumor suppressor. Biochim. Biophys. Acta 1470, M37–M53 10722926 10.1016/s0304-419x(99)00037-2

[2] Rice J. C., and Allis C. D. (2001) Histone methylation versus histone acetylation: New insights into epigenetic regulation. Curr. Opin. Cell Biol. 13, 263–273 10.1016/S0955-0674(00)00208-8 11343896

[3] Chicoine L. G., Richman R., Cook R. G., Gorovsky M. A., and Allis C. D. (1987) A single histone acetyltransferase from *Tetrahymena* macronuclei catalyzes deposition-related acetylation of free histones and transcription-related acetylation of nucleosomal histones. J. Cell Biol. 105, 127–135 10.1083/jcb.105.1.127 3611182 PMC2114890

[4] Deng Z., Chen C. J., Chamberlin M., Lu F., Blobel G. A., Speicher D., Cirillo L. A., Zaret K. S., and Lieberman P. M. (2003) The CBP bromodomain and nucleosome targeting are required for Zta-directed nucleosome acetylation and transcription activation. Mol. Cell Biol. 23, 2633–2644 10.1128/MCB.23.8.2633-2644.2003 12665567 PMC152567

[5] Lu F., Zhou J., Wiedmer A., Madden K., Yuan Y., and Lieberman P. M. (2003) Chromatin remodeling of the Kaposi's sarcoma-associated herpesvirus ORF50 promoter correlates with reactivation from latency. J. Virol. 77, 11425–11435 10.1128/JVI.77.21.11425-11435.2003 14557628 PMC229253

[6] Wooldridge T. R., and Laimins L. A. (2008) Regulation of human papillomavirus type 31 gene expression during the differentiation-dependent life cycle through histone modifications and transcription factor binding. Virology 374, 371–380 10.1016/j.virol.2007.12.011 18237759 PMC2410142

[7] Hancock M. H., Cliffe A. R., Knipe D. M., and Smiley J. R. (2010) Herpes simplex virus VP16, but not ICP0, is required to reduce histone occupancy and enhance histone acetylation on viral genomes in U2OS osteosarcoma cells. J. Virol. 84, 1366–1375 10.1128/JVI.01727-09 19939931 PMC2812316

[8] Ott M., Schnölzer M., Garnica J., Fischle W., Emiliani S., Rackwitz H. R., and Verdin E. (1999) Acetylation of the HIV-1 Tat protein by p300 is important for its transcriptional activity. Curr. Biol. 9, 1489–1492 10.1016/S0960-9822(00)80120-7 10607594

[9] Lu F., Day L., Gao S. J., and Lieberman P. M. (2006) Acetylation of the latency-associated nuclear antigen regulates repression of Kaposi's sarcoma-associated herpesvirus lytic transcription. J. Virol. 80, 5273–5282 10.1128/JVI.02541-05 16699007 PMC1472144

[10] Quinlan E. J., Culleton S. P., Wu S. Y., Chiang C. M., and Androphy E. J. (2013) Acetylation of conserved lysines in bovine papillomavirus E2 by p300. J. Virol. 87, 1497–1507 10.1128/JVI.02771-12 23152516 PMC3554136

[11] Mondal A., Potts G. K., Dawson A. R., Coon J. J., and Mehle A. (2015) Phosphorylation at the homotypic interface regulates nucleoprotein oligomerization and assembly of the influenza virus replication machinery. PLoS Pathog. 11, e1004826 10.1371/journal.ppat.1004826 25867750 PMC4395114

[12] Turrell L., Hutchinson E. C., Vreede F. T., and Fodor E. (2015) Regulation of influenza A virus nucleoprotein oligomerization by phosphorylation. J. Virol. 89, 1452–1455 10.1128/JVI.02332-14 25355893 PMC4300657

[13] Gabriel G., Herwig A., and Klenk H. D. (2008) Interaction of polymerase subunit PB2 and NP with importin α1 is a determinant of host range of influenza A virus. PLoS Pathog. 4, e11 10.1371/journal.ppat.0040011 18248089 PMC2222953

[14] Nakada R., Hirano H., and Matsuura Y. (2015) Structure of importin-α bound to a non-classical nuclear localization signal of the influenza A virus nucleoprotein. Sci. Rep. 5, 15055 10.1038/srep15055 26456934 PMC4601014

[15] Han Q., Chang C., Li L., Klenk C., Cheng J., Chen Y., Xia N., Shu Y., Chen Z., Gabriel G., Sun B., and Xu K. (2014) Sumoylation of influenza A virus nucleoprotein is essential for intracellular trafficking and virus growth. J. Virol. 88, 9379–9390 10.1128/JVI.00509-14 24920808 PMC4136286

[16] Zheng W., Li J., Wang S., Cao S., Jiang J., Chen C., Ding C., Qin C., Ye X., Gao G. F., and Liu W. (2015) Phosphorylation controls the nuclear-cytoplasmic shuttling of influenza A virus nucleoprotein. J. Virol. 89, 5822–5834 10.1128/JVI.00015-15 25787277 PMC4442427

[17] Liao T. L., Wu C. Y., Su W. C., Jeng K. S., and Lai M. M. (2010) Ubiquitination and deubiquitination of NP protein regulates influenza A virus RNA replication. EMBO J. 29, 3879–3890 10.1038/emboj.2010.250 20924359 PMC2989104

[18] Lin Y. C., Jeng K. S., and Lai M. M. C. (2017) CNOT4-mediated ubiquitination of influenza A virus nucleoprotein promotes viral RNA replication. mBio 8, e00597–17 10.1128/mBio.00597-17 28536288 PMC5442456

[19] Giese S., Ciminski K., Bolte H., Moreira É. A., Lakdawala S., Hu Z., David Q., Kolesnikova L., Götz V., Zhao Y., Dengjel J., Chin Y. E., Xu K., and Schwemmle M. (2017) Role of influenza A virus NP acetylation on viral growth and replication. Nat. Commun. 8, 1259 10.1038/s41467-017-01112-3 29097654 PMC5668263

[20] Marazzi I., Ho J. S., Kim J., Manicassamy B., Dewell S., Albrecht R. A., Seibert C. W., Schaefer U., Jeffrey K. L., Prinjha R. K., Lee K., García-Sastre A., Roeder R. G., and Tarakhovsky A. (2012) Suppression of the antiviral response by an influenza histone mimic. Nature 483, 428–433 10.1038/nature10892 22419161 PMC3598589

[21] Hatakeyama D., Shoji M., Yamayoshi S., Hirota T., Nagae M., Yanagisawa S., Nakano M., Ohmi N., Noda T., Kawaoka Y., and Kuzuhara T. (2014) A novel functional site in the PB2 subunit of influenza A virus essential for acetyl-CoA interaction, RNA polymerase activity, and viral replication. J. Biol. Chem. 289, 24980–24994 10.1074/jbc.M114.559708 25063805 PMC4155666

[22] Dyda F., Klein D. C., and Hickman A. B. (2000) GCN5-related N-acetyltransferases: a structural overview. Annu. Rev. Biophys. Biomol. Struct. 29, 81–103 10.1146/annurev.biophys.29.1.81 10940244 PMC4782277

[23] Sterner D. E., and Berger S. L. (2000) Acetylation of histones and transcription-related factors. Microbiol. Mol. Biol. Rev. 64, 435–459 10.1128/MMBR.64.2.435-459.2000 10839822 PMC98999

[24] Takechi S., and Nakayama T. (1999) Sas3 is a histone acetyltransferase and requires a zinc finger motif. Biochem. Biophys. Res. Commun. 266, 405–410 10.1006/bbrc.1999.1836 10600516

[25] Ghizzoni M., Wu J., Gao T., Haisma H. J., Dekker F. J., and George Zheng Y. (2012) 6-alkylsalicylates are selective Tip60 inhibitors and target the acetyl-CoA binding site. Eur. J. Med. Chem. 47, 337–344 10.1016/j.ejmech.2011.11.001 22100137 PMC3399519

[26] Balasubramanyam K., Swaminathan V., Ranganathan A., and Kundu T. K. (2003) Small molecule modulators of histone acetyltransferase p300. J. Biol. Chem. 278, 19134–19140 10.1074/jbc.M301580200 12624111

[27] Pereira J. M., Severino R. P., Vieira P. C., Fernandes J. B., da Silva M. F., Zottis A., Andricopulo A. D., Oliva G., and Corrêa A. G. (2008) Anacardic acid derivatives as inhibitors of glyceraldehyde-3-phosphate dehydrogenase from *Trypanosoma cruzi*. Bioorg. Med. Chem. 16, 8889–8895 10.1016/j.bmc.2008.08.057 18789702

[28] Modak R., Basha J., Bharathy N., Maity K., Mizar P., Bhat A. V., Vasudevan M., Rao V. K., Kok W. K., Natesh N., Taneja R., and Kundu T. K. (2013) Probing p300/CBP associated factor (PCAF)-dependent pathways with a small molecule inhibitor. ACS Chem. Biol. 8, 1311–1323 10.1021/cb4000597 23570531

[29] Balasubramanyam K., Altaf M., Varier R. A., Swaminathan V., Ravindran A., Sadhale P. P., and Kundu T. K. (2004) Polyisoprenylated benzophenone, garcinol, a natural histone acetyltransferase inhibitor, represses chromatin transcription and alters global gene expression. J. Biol. Chem. 279, 33716–33726 10.1074/jbc.M402839200 15155757

[30] Ye Q., Guu T. S., Mata D. A., Kuo R. L., Smith B., Krug R. M., and Tao Y. J. (2012) Biochemical and structural evidence in support of a coherent model for the formation of the double-helical influenza A virus ribonucleoprotein. mBio 4, e00467–12 10.1128/mBio.00467-12 23269829 PMC3531806

[31] Lau N. C., Kolkman A., van Schaik F. M., Mulder K. W., Pijnappel W. W., Heck A. J., and Timmers H. T. (2009) Human Ccr4-Not complexes contain variable deadenylase subunits. Biochem. J. 422, 443–453 10.1042/BJ20090500 19558367

[32] Zhang T., Cooper S., and Brockdorff N. (2015) The interplay of histone modifications—writers that read. EMBO Rep. 16, 1467–1481 10.15252/embr.201540945 26474904 PMC4641500

[33] Drazic A., Myklebust L. M., Ree R., and Arnesen T. (2016) The world of protein acetylation. Biochim. Biophys. Acta 1864, 1372–1401 10.1016/j.bbapap.2016.06.007 27296530

[34] Koutsogiannouli E. A., Wagner N., Hader C., Pinkerneil M., Hoffmann M. J., and Schulz W. A. (2017) Differential effects of histone acetyltransferase GCN5 or PCAF knockdown on urothelial carcinoma cells. Int. J. Mol. Sci. 18, 1449 10.3390/ijms18071449 28678170 PMC5535940

[35] Grant P. A., Duggan L., Côté J., Roberts S. M., Brownell J. E., Candau R., Ohba R., Owen-Hughes T., Allis C. D., Winston F., Berger S. L., and Workman J. L. (1997) Yeast Gcn5 functions in two multisubunit complexes to acetylate nucleosomal histones: Characterization of an Ada complex and the SAGA (Spt/Ada) complex. Genes Dev. 11, 1640–1650 10.1101/gad.11.13.1640 9224714

[36] McMahon S. B., Van Buskirk H. A., Dugan K. A., Copeland T. D., and Cole M. D. (1998) The novel ATM-related protein TRRAP is an essential cofactor for the c-Myc and E2F oncoproteins. Cell 94, 363–374 10.1016/S0092-8674(00)81479-8 9708738

[37] McMahon S. B., Wood M. A., and Cole M. D. (2000) The essential cofactor TRRAP recruits the histone acetyltransferase hGCN5 to c-Myc. Mol. Cell Biol. 20, 556–562 10.1128/MCB.20.2.556-562.2000 10611234 PMC85131

[38] Dang C. V. (1999) c-Myc target genes involved in cell growth, apoptosis, and metabolism. Mol. Cell Biol. 19, 1–11 10.1128/MCB.19.1.1 9858526 PMC83860

[39] Smallwood H. S., Duan S., Morfouace M., Rezinciuc S., Shulkin B. L., Shelat A., Zink E. E., Milasta S., Bajracharya R., Oluwaseum A. J., Roussel M. F., Green D. R., Pasa-Tolic L., and Thomas P. G. (2017) Targeting metabolic reprogramming by influenza infection for therapeutic intervention. Cell Rep. 19, 1640–1653 10.1016/j.celrep.2017.04.039 28538182 PMC5599215

[40] Shoemaker J. E., Fukuyama S., Eisfeld A. J., Muramoto Y., Watanabe S., Watanabe T., Matsuoka Y., Kitano H., and Kawaoka Y. (2012) Integrated network analysis reveals a novel role for the cell cycle in 2009 pandemic influenza virus-induced inflammation in macaque lungs. BMC Syst. Biol. 6, 117 10.1186/1752-0509-6-117 22937776 PMC3481363

[41] Liu L., Scolnick D. M., Trievel R. C., Zhang H. B., Marmorstein R., Halazonetis T. D., and Berger S. L. (1999) p53 sites acetylated *in vitro* by PCAF and p300 are acetylated in vivo in response to DNA damage. Mol. Cell Biol. 19, 1202–1209 10.1128/MCB.19.2.1202 9891054 PMC116049

[42] Nailwal H., Sharma S., Mayank A. K., and Lal S. K. (2015) The nucleoprotein of influenza A virus induces p53 signaling and apoptosis via attenuation of host ubiquitin ligase RNF43. Cell Death Dis. 6, e1768 10.1038/cddis.2015.131 25996295 PMC4669709

[43] Yan W., Wei J., Deng X., Shi Z., Zhu Z., Shao D., Li B., Wang S., Tong G., and Ma Z. (2015) Transcriptional analysis of immune-related gene expression in p53-deficient mice with increased susceptibility to influenza A virus infection. BMC Med. Genomics 8, 52 10.1186/s12920-015-0127-8 26282854 PMC4539693

[44] Chang M. F., Baker S. C., Soe L. H., Kamahora T., Keck J. G., Makino S., Govindarajan S., and Lai M. M. (1988) Human hepatitis delta antigen is a nuclear phosphoprotein with RNA-binding activity. J. Virol. 62, 2403–2410 3373572 10.1128/jvi.62.7.2403-2410.1988 PMC253398

[45] Mu J. J., Tsay Y. G., Juan L. J., Fu T. F., Huang W. H., Chen D. S., and Chen P. J. (2004) The small delta antigen of hepatitis delta virus is an acetylated protein and acetylation of lysine 72 may influence its cellular localization and viral RNA synthesis. Virology 319, 60–70 10.1016/j.virol.2003.10.024 14967488

[46] Watanabe T., Kawakami E., Shoemaker J. E., Lopes T. J., Matsuoka Y., Tomita Y., Kozuka-Hata H., Gorai T., Kuwahara T., Takeda E., Nagata A., Takano R., Kiso M., Yamashita M., Sakai-Tagawa Y., Katsura H., Nonaka N., Fujii H., Fujii K., Sugita Y., Noda T., Goto H., Fukuyama S., Watanabe S., Neumann G., Oyama M., Kitano H., and Kawaoka Y. (2014) Influenza virus-host interactome screen as a platform for antiviral drug development. Cell Host Microbe 16, 795–805 10.1016/j.chom.2014.11.002 25464832 PMC4451456

[47] Dhalluin C., Carlson J. E., Zeng L., He C., Aggarwal A. K., and Zhou M. M. (1999) Structure and ligand of a histone acetyltransferase bromodomain. Nature 399, 491–496 10.1038/20974 10365964

[48] Hohmann A. F., and Vakoc C. R. (2014) A rationale to target the SWI/SNF complex for cancer therapy. Trends Genet. 30, 356–363 10.1016/j.tig.2014.05.001 24932742 PMC4112150

[49] Dornfeld D., Dudek A. H., Vausselin T., Günther S. C., Hultquist J. F., Giese S., Khokhlova-Cubberley D., Chew Y. C., Krogan N. J., Garcia-Sastre A., Schwemmle M., and Shaw M. L. (2018) SMARCA2-regulated host cell factors are required for MxA restriction of influenza A viruses. Sci. Rep. 8, 2092 10.1038/s41598-018-20458-2 29391557 PMC5794779

[50] Clements A., Rojas J. R., Trievel R. C., Wang L., Berger S. L., and Marmorstein R. (1999) Crystal structure of the histone acetyltransferase domain of the human PCAF transcriptional regulator bound to coenzyme A. EMBO J. 18, 3521–3532 10.1093/emboj/18.13.3521 10393169 PMC1171431

[51] Sugita Y., Sagara H., Noda T., and Kawaoka Y. (2013) Configuration of viral ribonucleoprotein complexes within the influenza A virion. J. Virol. 87, 12879–12884 10.1128/JVI.02096-13 24067952 PMC3838143

[52] Sadaie M., Shinmyozu K., and Nakayama J. (2008) A conserved SET domain methyltransferase, Set11, modifies ribosomal protein Rpl12 in fission yeast. J. Biol. Chem. 283, 7185–7195 10.1074/jbc.M709429200 18195021

[53] Ozawa M., Fujii K., Muramoto Y., Yamada S., Yamayoshi S., Takada A., Goto H., Horimoto T., and Kawaoka Y. (2007) Contributions of two nuclear localization signals of influenza A virus nucleoprotein to viral replication. J. Virol. 81, 30–41 10.1128/JVI.01434-06 17050598 PMC1797272

[54] Murakami S., Horimoto T., Yamada S., Kakugawa S., Goto H., and Kawaoka Y. (2008) Establishment of canine RNA polymerase I-driven reverse genetics for influenza A virus: its application for H5N1 vaccine production. J. Virol. 82, 1605–1609 10.1128/JVI.0187-07 18045936 PMC2224466

[55] Shoji M., Arakaki Y., Esumi T., Kohnomi S., Yamamoto C., Suzuki Y., Takahashi E., Konishi S., Kido H., and Kuzuhara T. (2015) Bakuchiol is a phenolic isoprenoid with novel enantiomer-selective anti-influenza A virus activity involving Nrf2 activation. J. Biol. Chem. 290, 28001–28017 10.1074/jbc.M115.669465 26446794 PMC4646038

